# Mapping the Research Landscape of Intra-Articular Knee Injections: A Bibliometric Analysis Using the Scopus Database

**DOI:** 10.7759/cureus.65647

**Published:** 2024-07-29

**Authors:** Abdullah Aqeel Musa, Chooi Leng Low, Khairul Nizam Siron, Mohamad Hilmi Mohamad Nazarallah, Ren Yi Kow, Intan Bazilah Abu Bakar, Aidi Aswadi Halim Lim, Ahmad Hafiz Zulkifly

**Affiliations:** 1 Orthopaedics and Trauma, Tengku Ampuan Rahimah Hospital, Klang, MYS; 2 Department of Radiology, International Islamic University Malaysia, Kuantan, MYS; 3 Department of Orthopaedics, Traumatology & Rehabilitation, International Islamic University Malaysia, Kuantan, MYS; 4 Orthopaedic Department, Universiti Sains Islam Malaysia, Nilai, MYS

**Keywords:** review article, bibliometric analyis, intra articular injection, knee osteoarthritis/ koa, knee injury and osteoarthritis outcome score, osteoarthritis (oa)

## Abstract

Intraarticular injection of osteoarthritis knee is one of the treatment options for pain management and delays the need for knee surgery. Various materials have been promoted for the procedure, ranging from corticosteroid to viscosupplement to the more recent autologous biological materials. Despite the increasing attention and interest in regard to the material selection, efficacy, safety, and effect of this intervention, a comprehensive bibliometric analysis using the Scopus database has yet to be conducted. In this bibliometric analysis, we reviewed the Scopus database from 2003 to 2023 to investigate the literature on intraarticular injection for the treatment of knee osteoarthritis. A total of 1,318 articles that satisfied the selection criteria were included in this review. The trend of intervention shows changes since 2006, with corticosteroid injection and hyaluronic acid as the main topics of publication before 2006. However, starting in 2010, there has been a noticeable shift towards biological agents, such as plasma-rich proteins, and autologous materials, including marrow aspiration and stromal vascular fraction. This shift reflects the increasing interest in regenerative medicine and the potential of these newer therapies to provide improved outcomes. The overwhelming majority of the articles were authored by researchers and clinicians from across European countries, the United States of America (USA), and Australia. Similarly, most of the articles with the highest number of citations were authored by researchers and clinicians from these regions. This comprehensive bibliometric analysis using Scopus in the domain of intraarticular injection has the potential to act as a roadmap for researchers, clinicians, and policymakers, facilitating informed decision-making, promoting collaborative initiatives, and guiding the development of future studies to further advance the options of knee intraarticular injection, specifically in the management of knee osteoarthritis.

## Introduction and background

Primary knee osteoarthritis is a degenerative joint disease typically resulting from wear and tear, leading to a progressive loss of articular cartilage [1-3]. It is a chronic, progressive disorder that affects the entire joint, including bone and cartilage and is characterized by variable inflammation, subchondral bone structural changes, and damage to the articular cartilage [4-6]. The intensity and rate of progression of symptoms can vary among individuals; however, symptoms generally become more severe, debilitating, and frequent over time [7-9]. Common symptoms include knee pain that gradually worsens with activity, stiffness, swelling, and mechanical pain that worsens over time [10-12].

Intraarticular injection of the knee is one treatment option, particularly for patients who do not tolerate oral pharmacological therapy or who wish to delay the need for surgical treatment. It has been shown that intraarticular injections can provide short-term relief from symptoms and may improve pain and function [1,2]. Multiple modalities have been proposed for these injections, including corticosteroid injections, hyaluronic acid, biologic agents such as platelet-rich plasma, stem cells, and marrow aspiration, each with its own proposed role and benefits [1,2].

With the advancement of open-access data mining, search engines such as Google Trends make reviews much more convenient and efficient [13,14]. Similarly, bibliometric analysis using databases like PubMed, Scopus, and the Web of Science Core Collection is becoming easier for researchers [15-19]. Bibliometric analysis is an effective and widely used approach for evaluating the scholarly impact of published literature and analyzing large volumes of scientific data. It summarizes research interests, assesses relationships within a research field, and evaluates academic quality and impact by measuring the number of publications and citations in each reviewed article. Additionally, bibliometric analysis uncovers collaborations among authors, institutions, and countries, which is crucial for forming new collaborative projects with researchers of similar expertise. It also identifies research gaps within subjects, providing valuable insights and guidance to researchers and institutions. Thus far, bibliometric analysis of intraarticular injection for knee osteoarthritis has been conducted using the Web of Science Core Collection but not the Scopus database [2]. This review aims to address that knowledge gap by evaluating publications on intraarticular injection for knee osteoarthritis through the lens of the Scopus database.

## Review

Search strategy

The literature on intraarticular injection was sourced from the Scopus database. The search strategy was formulated using the keywords ‘(knee) AND (osteoarthritis OR arthritis) AND (injection OR intraarticular OR intraarticular AND injection).’ The search covered the period from January 2003 to December 2023. The retrieved articles were then screened by the two principal authors for suitability. Only clinical studies (animal studies were excluded) related to intraarticular injection of the knee were included. Literature pertaining to injections of other synovial joints, such as shoulder and hip joints, was excluded. Similarly, other types of publications, such as book chapters, letters, trade journals, and errata, were also excluded.

The screened articles were imported into R Studio 2021 for Windows with the ‘bibliometrix’ package installed in R. Information such as authorship, title, publication year, number of citations, affiliations, journal source, references, and keywords were extracted using the R software. The data presentation, including illustrative graphs and tables, was created using Microsoft Excel 2019.

Results

**Figure 1:**
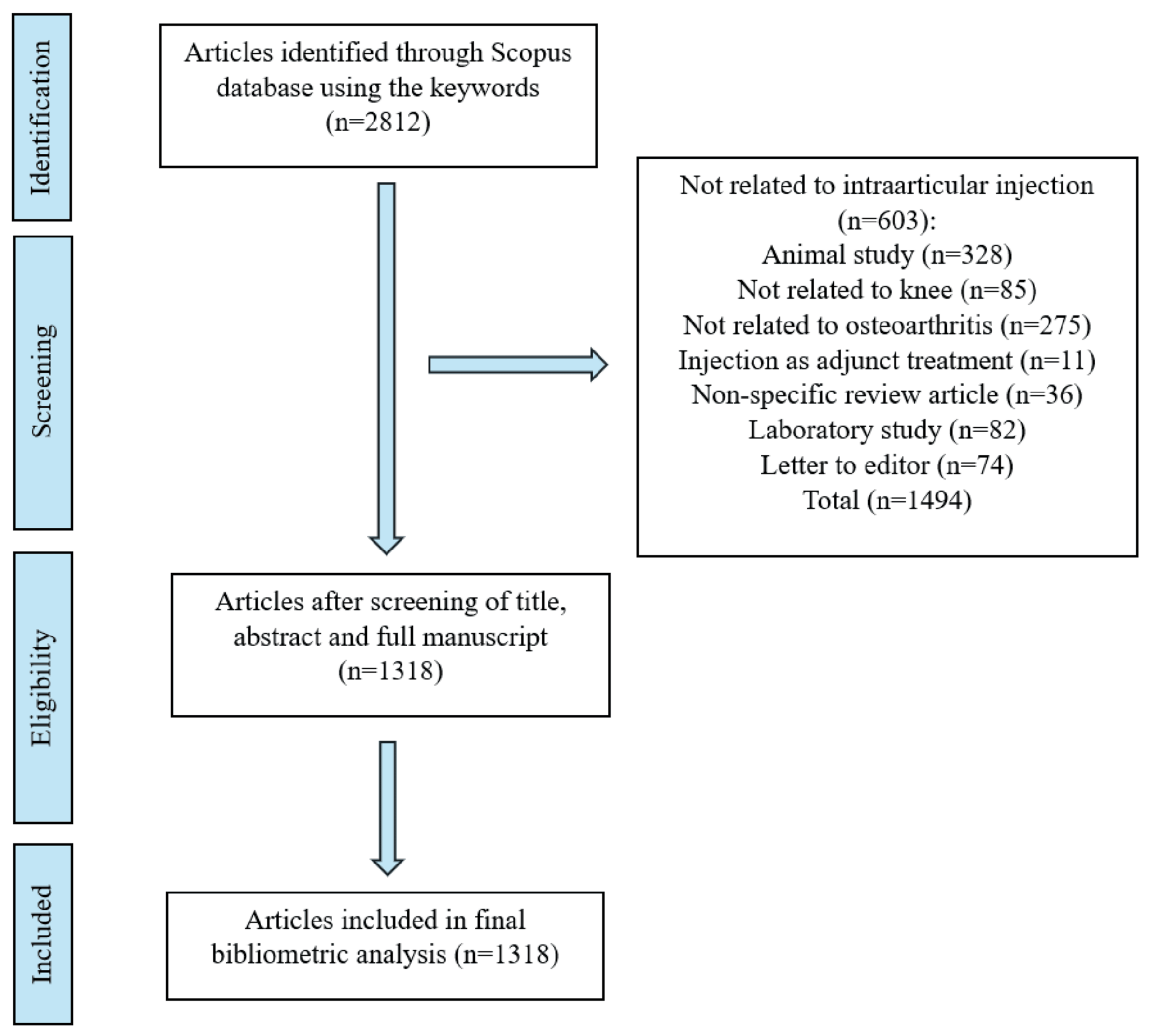
Annual publications on knee intra-articular injection

A total of 2,812 articles were retrieved from the Scopus database for the years 2003-2023 using the search terms presented. After screening by the lead author, only 1,318 articles met the inclusion criteria and were analyzed for the review (Figure 1).

**Figure 2:**
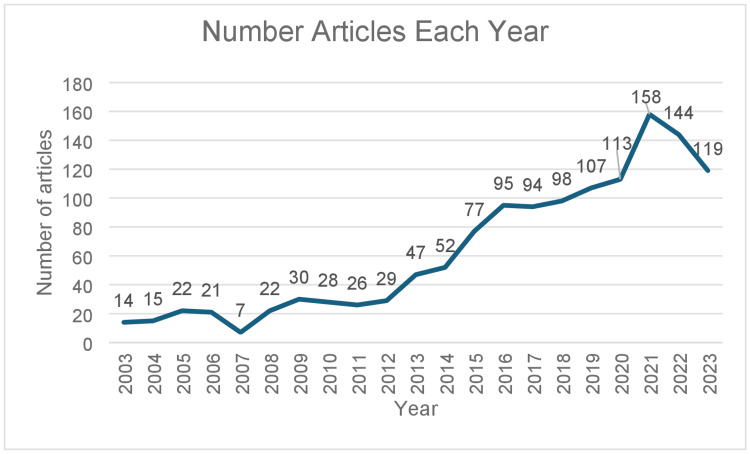
Publications related to intraarticular injection over the years

As depicted in Figure 2, there has been increasing interest in the intervention of intraarticular injection for knee osteoarthritis, with a significant rise in the number of publications starting in 2014. The number of publications related to this intervention shows a steady increase, reaching a peak of 158 publications in 2021. Despite a declining trend in 2022 and 2023, the number of publications on this topic remains high, indicating continued interest in this intervention.

A total of 1,318 articles were published in various journals within this study period of 2003-2023. Table 1 shows the top 10 journals with the highest number of publications on intraarticular injection of knee osteoarthritis. BMC musculoskeletal disorder leads the table with 47 publications, followed by Osteoarthritis and Cartilage (43 publications), Knee Surgery, Sports Traumatology, Arthroscopy (37 publications), American Journal of Sports Medicine (37 publications), American Journal of Sports Medicine (36 publications), Arthroscopy - Journal of Arthroscopic and Related Surgery (35 publications), Clinical Rheumatology (30 publications), Cartilage (29 publications), Journal of Arthroplasty (21 publications), Medicine United States (21 publications), and International Orthopedic (18 publications).

**Table 1:** The top 10 journals with the highest number of articles

Journal Name	Number of articles	Percentage
BMC musculoskeletal disorder	46	3.5
Osteoarthritis and cartilage	39	3.0
Knee Surgery, sports traumatology, arthroscopy	36	2.7
American Journal of Sports Medicine	36	2.7
Arthroscopy – journal of arthroscopic and related surgery	34	2.6
Cartilage	29	2.2
Clinical Rheumatology	30	2.3
Journal of Arthroplasty	21	1.6
Medicine (United States of America)	21	1.6
International Orthopaedic	18	1.4

Table 2 summarizes the top 10 authors with the highest number of articles in the study of knee osteoarthritis intraarticular injection. Kon E had the highest number of publications, with a total of 25, followed by Chevalier X et al. (21 publications), Conrozier T (20 publications), McAlindon TE et al. (19 publications), Altman RD (18 publications), Hunter DJ (17 publications), Di Matteo B (16 publications), and Cole BJ and Migliore A (14 publications). 

**Table 2:** Top 10 authors with the highest number of publications

Author’s Name	Number of articles	Percentage
Filardo G	25	1.9
Kon E	25	1.9
Chevalier X	21	1.6
Conrozier T	20	1.5
Mcalindon TE	19	1.4
Altman RD	18	1.4
Hunter DJ	17	1.3
Di Matteo B	16	1.2
Cole BJ	14	1.1
Migliore A	14	1.1

Table 3 summarizes the institutions with the highest number of publications. Rush University Medical Centre leads, and Humanitas University produced 58 articles, followed by Rizzoli Orthopaedic Institute (49 articles), Shahid Beheshti University of Medical Science (41 articles), University of California (39 articles), Clinica Universidad de Navarra (38 articles), Royan Institute for Stem Cell Biology and Technology (36 articles), University of Sydney (32 articles), Bologna (31 articles), and Peking University People’s Hospital (29 articles). 

**Table 3:** The top 10 institutions with the highest number of publications

Institutions	Number of articles	Percentage
Rush University Medical Centre	58	4.4
Humanitas University	58	4.4
Rizzoli Orthopaedic Institute	49	3.7
Shahid Beheshti University of Medical Science	41	3.1
University of California	39	3.0
Clinica Universidad de Navarra	38	2.9
Royan Institute for Stem Cell Biology and Technology	36	2.7
University of Sydney	32	2.4
Bologna	31	2.4
Peking University People’s Hospital	29	2.2

Table 4 displays the top 10 countries with the highest number of publications. The USA produced the highest number of publications with 273 articles, followed by China with 153 articles, Italy with 109 articles, France with 65 articles, Turkey with 49 articles, Spain with 37 articles, the United Kingdom with 41 articles, Korea with 36 articles, Japan with 32 articles, and Iran with 29 articles. Among the top countries, only France and the United Kingdom had a multiple-country production ratio of more than 0.2 (20%).

**Table 4:** Corresponding author’s country with the highest number of publications SCP: single country production; MCP: multiple country production

Country	Number of articles	SCP	MCP	MCP Ratio
United States of America	273	221	52	0.190
China	153	139	14	0.092
Italy	109	89	20	0.183
France	65	42	23	0.354
Turkey	49	48	1	0.020
United Kingdom	41	29	12	0.293
Spain	37	32	5	0.135
Korea	36	32	4	0.111
Japan	32	28	4	0.125
Iran	29	24	5	0.172

Table 5 evidences that the intraarticular injection type with hyaluronic acid is the most commonly studied with 469 studies, followed by platelet-rich plasma (258 studies), steroid (234 studies), mesenchymal stem cell (102 studies), prolotherapy (33 studies), bone marrow aspiration concentrate (23 studies), autologous stromal vascular fraction (18 studies), botulinum toxin (16 studies), adipose-derived stromal cell (16 studies), microfragmented adipose tissue (14 studies), and ozone (13 studies). 

**Table 5:** Type of intraarticular injection

Type of Intraarticular injection	Number	Percentage
Hyaluronic Acid	469	35.5
Plasma Rich Protein	258	19.6
Corticosteroid	234	17.7
Mesenchymal Stem Cell	102	7.7
Prolotherapy	33	2.5
Bone Marrow Aspiration Concentrate	23	1.7
Autologous Stromal Vascular Fraction	18	1.4
Autologous condition serum	16	1.2
Adipose derived stromal cell	16	1.2
Botulinum toxin	16	1.2
Microfragmented adipose tissue	14	1.1
Ozone	13	0.9
Polynucleotide	8	0.6
Plasma rich growth factor	6	0.4
Sprifermin	6	0.4
Autologous protein solution	5	0.4
Autologous adipose tissue	5	0.4
Saline	4	0.3
Chitosan	4	0.3
Interleukin 1 receptor blocking agent	4	0.3
Ketorolak	3	0.2
Diclofenac	3	0.2
Low Molecular Weight Fraction of 5% human serum Albumin	3	0.2
Autologous conditioned plasma	2	0.2
Amniotic Suspension Allograft	2	0.2
Collagen	2	0.2
Amniotic stem cell	2	0.2
Adipose derived stem cell	2	0.2
Clodronate	2	0.2
Bone morphogenetic protein-7	2	0.2
Lidocaine	2	0.2
Adalimumab	2	0.2
Autologous protein solution	2	0.2
Lorecivivint	2	0.2
Umbilical cord-derived Wharton’s jelly	2	0.2
Placenta derived product	1	0.1
Capsaicin	1	0.1
Tumor necrosis factor antagonist	1	0.1
Deproteinized hemodialsate	1	0.1
Glucosamine HCL	1	0.1
Peripheral blood-derived mononuclear cells	1	0.1
Autologous subcutaneous adipose tissue	1	0.1
Epigallocatechin	1	0.1
Allogenic Umbilical Cord Tissue	1	0.1
Adipose-derived regenerative cells	1	0.1
Rapamycin	1	0.1
Tenoxicam	1	0.1
Pentosan polysulfate	1	0.1
Amniotic stem cell	1	0.1
Amnion chorion membrane	1	0.1
Umbilical allogenic platelet concentrate	1	0.1
Amniotic Membrane/Umbilical Cord Particulate	1	0.1
Platelet derived factor	1	0.1
Tropomyosin receptor kinase A inhibitor	1	0.1
Autologous condition plasma	1	0.1
Polymerize collagen	1	0.1
Amniotic membrane particulate	1	0.1
Bioactive cell free formulation	1	0.1
Microfragment lipoaspirate	1	0.1
Marrow derives mononuclear cell	1	0.1
Parecoxib	1	0.1
Adipose mesenchymal progenitor cell	1	0.1
Progenza	1	0.1
Placebo	1	0.1
Wnt pathway inhibitor	1	0.1
Autologous plasma	1	0.1
Human meniscus stem/progenitor cells	1	0.1

Table 6 shows the articles with the greatest number of citations. An article entitled ‘Viscosupplementation for the Treatment of Osteoarthritis of the Knee’ by Bellamy N et al. is the most cited article according to our analysis, with a total of 761 citations [20]. This was followed by an article by Jo CH et al. of Korea titled ‘Intraarticular Injection of Mesenchymal Stem Cells for the Treatment of Osteoarthritis of the Knee: A Proof of Clinical Trial’, which came in as the second most cited article with 667 citations [21]. Bellamy N et al.’s article ‘Intraarticular Corticosteroid for Treatment of Osteoarthritis of the Knee’ from 2005 came in third place (576 citations) [22]. This was followed by McAlindon TE et al.’s article, ‘Effect of Intraarticular Triamcinolone vs. Saline on Knee Cartilage Volume and Pain in Patients with Knee Osteoarthritis: A Randomized Controlled Trial' (506 citations), Bannuru RR et al.’s article ‘Comparative Effectiveness Pharmacologic Intervention for Knee Osteoarthritis: A Systematic Review and Network Meta-Analysis’ (464 citations), Moreland LW et al.’s ‘Intra-articular Hyaluronan (Hyaluronic acid) and Hylans for the Treatment of Osteoarthritis: Mechanisms of Action’, Vega A et al.’s ‘Treatment of Knee Osteoarthritis with Allogeneic Bone Marrow Mesenchymal Stem Cells: A Randomized Controlled Trial’, Raynauld JP et al.’s ‘Safety and Efficacy of Long-term Intraarticular Steroid Injections in Osteoarthritis of the Knee: A Randomized, Double-blind, Placebo-controlled Trial’, Kon E et al.’s ‘Platelet-rich Plasma: Intra-articular Knee Injections Produced Favorable Results on Degenerative Cartilage Lesions’ and Chevalier X et al.’s ‘Intraarticular Injection of Anakinra in Osteoarthritis of the Knee: A Multicenter, Randomized, Double-blind, Placebo-controlled Study’ [23-29].

**Table 6:** Top 10 articles with the most citations RCT: Randomized control trial

Citation	Mean Citation	First Author	Country	Title	Year	Journal	Type
761	40.05	Bellamy N [20]	Australia	Viscosupplementation for the Treatment of Osteoarthritis of the Knee	2006	Cochrane database	Systematic review
667	60.64	Jo CH [21]	Korea	Intraarticular Injection of Mesenchymal Stem Cells for the Treatment of Osteoarthritis of the Knee: A Proof of Clinical Trial’	2014	Stem Cell	Clinical Trial
576	28.80	Bellamy N [22]	Australia	Intraarticular Corticosteroid for Treatment of Osteoarthritis of the Knee.	2005	Cochrane Database	Systematic review
506	63.25	McAlindon TE [23]	United States of America	Effect of Intra-articular Triamcinolone vs Saline on Knee Cartilage Volume and Pain in Patients with Knee Osteoarthritis: A Randomized Clinical Trial	2017	JAMA	RCT
464	46.40	Bannuru RR [24]	United States of America	Comparative Effectiveness of Pharmacologic Interventions for Knee Osteoarthritis: A Systematic Review and Network Meta-analysis	2015	ANN intern med	Meta analysis
464	21.09	Moreland LW [25]	United States of America	Intra-articular Hyaluronan (Hyaluronic acid) and Hylans for the Treatment of Osteoarthritis: Mechanisms of Action	2003	Arthritis Res Ther	Systematic review
430	43.00	Vega A [26]	Spain	Treatment of Knee Osteoarthritis with Allogeneic Bone Marrow Mesenchymal Stem Cells: A Randomized Controlled Trial	2015	Transplantation	RCT
424	19.27	Raynauld JP [27]	United Kingdom	Safety and Efficacy of Long-term Intraarticular Steroid Injections in Osteoarthritis of the Knee: A Randomized, Double-blind, Placebo-controlled Trial	2003	Arthritis Rheum	RCT
420	28.00	Kon E [28]	Italy	Platelet-rich Plasma: Intra-articular Knee Injections Produced Favorable Results on Degenerative Cartilage Lesions	2010	Knee surg sports traumatol arthroscopy	Prospective study
411	25.69	Chevalier X [29]	France	Intraarticular Injection of Anakinra in Osteoarthritis of the Knee: A Multicenter, Randomized, Double-blind, Placebo-controlled Study	2009	Arthritis Care and Research	RCT

**Figure 3:**
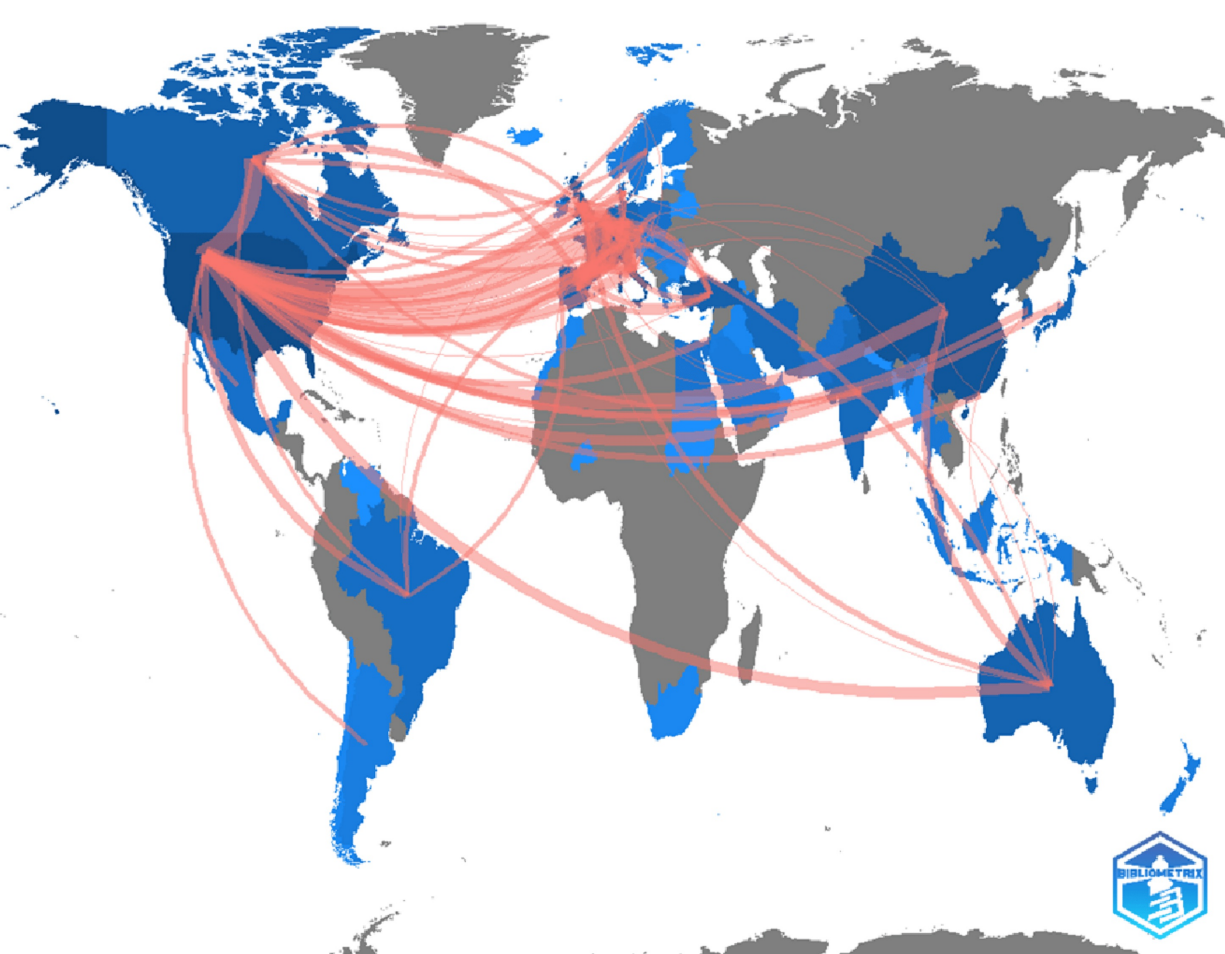
Countries' collaboration world map The pink lines depict the collaboration between countries, with the thickness of the pink line indicating the degree of collaboration. Note: Figure generated using the bibliometrix software in R Studio (R Studio: Integrated Development for R, Boston, USA [19].

Figure 3 shows countries’ collaborations on a world map. Compared to bibliometric analyses of other fields, the research on intraarticular injection of the knee demonstrated extensive multinational collaborations with numerous countries. Table 7 shows the top 10 collaboration partnerships, with the highest number of collaborations coming from the US-Canada partnership with 28 publications. This was followed by collaborations between the US and UK, with 25 publications. France-Belgium collaborations came in third with a total of 20 publications, followed by Italy-France collaborations with 17 publications. US collaborations with China and France and collaborations between Italy and the UK came next with 15 publications each, followed by France-UK collaborations with 14. There were 13 publication collaborations between France and Germany and between the US and Germany, accounting for ninth and tenth positions in the highest number of publication collaborations between countries. This indicates a global and interconnected approach to the researchers’ work, emphasizing the importance of international collaboration in the advancement of research and the exchange of knowledge.

**Table 7:** Most number of publication collaborations between countries

Country collaboration	Frequency
United States of America – Canada	28
United States of America – United Kingdom	25
France – Belgium	20
Italy – France	17
United States of America - China	15
United States of America – France	15
Italy – United Kingdom	15
France – United Kingdom	14
France – Germany	13
United States of America – Germany	13

Discussions

In this bibliometric analysis, we investigated intraarticular injections for knee osteoarthritis within the Scopus database. This review validates a wide range of pertinent information regarding the authors, topics, and time periods that have had a profound impact on the field of intraarticular injection for knee osteoarthritis. The trend in intraarticular injections for knee osteoarthritis has shown noticeable changes since 2006. Before 2006, the main topics of publication were mostly related to corticosteroid injections and hyaluronic acid. Nevertheless, starting in 2010, there has been a shift in trend towards biological agents, such as plasma-rich protein (PRP) and autologous materials, including marrow aspiration and stromal vascular fraction (SVF). This shift reflects the increasing interest in regenerative medicine and the potential of these newer injection modalities to provide improved outcomes. These newer therapies utilize a patient's own cells to promote tissue repair and regeneration, offering a more personalized treatment approach.

In this review, we identified the 10 most cited articles in the Scopus database related to intraarticular injection for knee osteoarthritis. Citation analysis, despite its limitations, provides an objective measure of peer recognition and offers insights into the readership of the articles. It is crucial for researchers to acknowledge that citation rates alone may not fully capture the multifaceted nature of research impact. Though not directly linked to study quality, articles with high citation numbers indicate that various researchers have found the content beneficial and its material worthy of inclusion and discussion in their work.

The highest citation counts predominantly come from systematic review articles, with a significant one published in 2006 and a newer version in 2021. These reviews reflect the evolving focus of research on multiple treatment modalities for intraarticular injections. Before 2006, hyaluronic acid and corticosteroids were the main themes of the study. However, a noticeable trend began after 2006, with increasing research on plasma-rich protein injections, mesenchymal stem cells, autologous stromal vascular fraction, bone marrow aspiration, and other biological modalities [20-29].

Researchers from the USA and European countries exhibit a significant trend of publishing more articles and receiving higher citations. Authors from these regions hold a substantial share in this field, underscoring their significant contribution to the global research landscape. However, the field of intraarticular injection shows considerable potential, with articles published across the globe, including Australia, Asian countries, European countries, and the United States.

The variety of materials used for intraarticular injections has also expanded. Initially, steroid injections were common, followed by hyaluronic acid as the preferred viscosupplementation. More recently, there has been increased interest in biological materials such as plasma-rich proteins, bone marrow aspirates, autologous stromal vascular fractions, and even placenta-derived materials.

Limitations

There are several intrinsic limitations to this bibliometric analysis. First, selection bias is present in that only English articles derived from the Scopus database are included. Second, due to the time frame of the literature search, the number of citations received by the articles reviewed in this analysis may be potentially higher for articles published in recent years. The long time span for publications not only affects the number of citations but also introduces variation in the authors’ affiliations because clinicians and researchers may have moved to different institutions or countries. Additionally, because only the Scopus database was examined in this bibliometric analysis, the citation count may differ if other databases, such as Web of Science Core Collection or Google Scholar, are used.

## Conclusions

Intra-articular injection for knee osteoarthritis is a popular procedure that continues to attract growing interest in the management of osteoarthritis. Research is ongoing to explore various materials for intervention and their potential to prevent the progression of osteoarthritis. This review offers a detailed account and bibliometric analysis of publications over a diverse range of durations. These findings provide insights into the status and research trends, offering valuable guidance for future practices and directions. Despite publications by researchers and clinicians from various countries, certain parts of the world had a higher scholarly impact in this field.
